# Resistance of Concrete with Various Types of Coarse Aggregate to Coupled Effects of Thermal Shocks and Chemicals

**DOI:** 10.3390/ma17040791

**Published:** 2024-02-06

**Authors:** Muhammad Monowar Hossain, Safat Al-Deen, Sukanta Kumer Shill, Md Kamrul Hassan

**Affiliations:** 1 School of Engineering and Information Technology, University of New South Wales, Sydney, NSW 2052, Australias.al-deen@unsw.edu.au (S.A.-D.);; 2 Department of Civil Engineering, Military Institute of Science and Technology, Dhaka 1216, Bangladesh

**Keywords:** lightweight aggregate, spalling, residual strength, thermal properties, microstructure

## Abstract

Rigid pavements at military airfields experience surface deterioration within 6–18 months of construction. The cause of this degradation is mainly due to combined exposure to repeated heat shocks from jet engine exhaust and spilled aviation oils (hydrocarbons). Surface degradation occurs in the form of disintegration of aggregates and cement paste into small pieces that pose severe risks of physical injury to maintenance crews or damage to an aircraft engine. Since coarse aggregates typically occupy 60–80% of the concrete volume, aggregates’ thermal properties and microstructure should play a crucial role in the degrading mechanism. At high temperatures, concrete with lightweight aggregates is reported to have better performance compared to concrete with normal-weight aggregate. Thus, the present study carried out a detailed investigation of the mechanical and thermal performance of lightweight aggregate concrete exposed to the combined effects of high temperatures and hydrocarbon oils simultaneously. To replicate harsh airfield operating conditions, standard-sized concrete cylinders were exposed to elevated temperatures using an electric oven. Additionally, a mixture of equal parts of aircraft engine oil, hydraulic oil, and kerosene was applied before each exposure to high temperatures. To identify the resistance of different concrete with various lightweight coarse aggregates, pumice, perlite, lytag (sintered fly ash), and crushed brick were used as lightweight coarse aggregates in concrete. Also, basalt aggregate concrete was used as a reference. After curing, cylinders were tested for the ultimate strength. Later, after every 20 cyclic exposures, three cylinders from each aggregate type were tested for residual comprehensive strength, thermal, chemical, and microstructural (SEM) properties. Overall, concrete with crushed brick aggregate and lytag used in this study showed superior resistance to the simulated airfield conditions. The findings of this study will provide valuable insights to select an appropriate coarse aggregate type for military airfield pavement construction, aiming to effectively minimize surface spalling.

## 1. Introduction

In concrete technology, coarse aggregates with a bulk density lower than 2000 kg/m^3^ are usually considered lightweight aggregates (LWA) [1]. Nowadays, LWA are an essential construction material to produce lightweight concrete for many applications in civil engineering industries. To produce lightweight aggregate concrete (LWAC), lightweight fine aggregate and lightweight coarse aggregate could be used combined, or normal fine aggregate could be used with LWA [2,3,4].

There are two types of LWA: artificial and natural. Rapid cooling of volcanic magma forms natural LWA; thus, the appearance and internal pore structure vary, making it difficult to control quality. A few examples of natural LWA include pumice, volcanic scoria, diatomite, sawdust, bottom ash, and starch-based aggregate. In addition, artificially made LWA include perlite, slate, lytag (sintered fly ash), expanded shale, vermiculite, and bonded fly ash. Crushed bricks are also used as LWA, since they resemble sintered clay aggregate and possess better thermal properties [5]. Since natural LWA are found in abundance in some regions of the world, their use in LWAC can significantly reduce the cost [6,7].

The key characteristics of LWA are low density, higher porosity, good thermal properties, and better fire resistance [3,8,9,10,11]. As stated, LWAC used in structural elements has inherent benefits of lower thermal coefficient and thermal expansion, higher strength-to-weight ratio, and better tensile strain capacity due to air voids in the LWA [1,12]. Over the last few decades, many countries adopted LWAC to build bridges and houses, such as the Prudential Life Building (42-story) in Chicago, the 50-story Australia Square Tower, large-span bridges in Norway, and an airfield pavement project at Philadelphia International Airport is using approximately 90,000 cu yd of foamed glass aggregate.

Fire’s adverse effect on concrete’s mechanical properties has been extensively studied throughout the 20th century [13,14]. Although aggregate is an inert material, it occupies 60–80% of the volume and influences the thermal, dimensional stability and elastic properties of concrete. The compressive strengths of the LWA highly influences the compressive strength of LWAC. Mineralogical properties of aggregate, such as shape, texture, moisture content, specific gravity and bulk unit weight also influence concrete’s mechanical properties. Thus, aggregate has a tangible impact on the phenomena once exposed to higher temperatures [15]. In the past, many researchers suggested that the addition of supplementary binding materials, such as fly ash, slag, silica fume, and metakaolin, increases the durability of LWAC [16]. However, a few other research studies indicated that silica fume or other supplementary materials might enhance concrete density, resulting in explosive spalling due to increased pore pressure at elevated temperatures [13].

**Figure 1:**
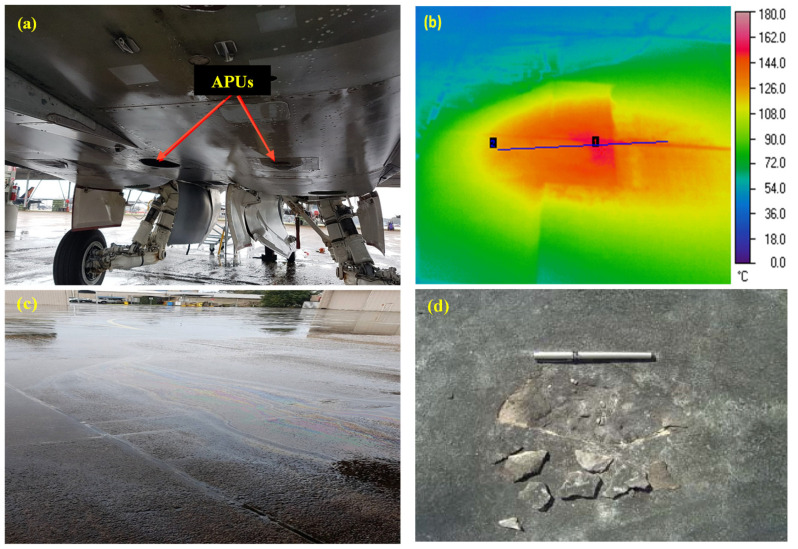
(**a**) APUs of F/A-18 [17]. (**b**) Heat map of concrete pavement exposed to APU exhaust. (The blue line connects Pt 1 and Pt 2, representing the centre and outer extent of exhaust impact area). (**c**) Pavement soaked with oils after single maintenance. (**d**) Spalling of concrete pavement [17].

Rigid pavements at military airbases are usually constructed using Portland cement concrete (PCC) [17]. Rigid pavements at military airbases face extremely harsh operating conditions [17,18]. In the early 1980s, after the induction of F/A-18 aircraft, rigid pavement has been experiencing rapid pavement surface damage issues worldwide [17,19]. Modern military aircraft’s hot exhaust from auxiliary power units (APUs) and aviation oil spills, either in conjunction or separately, affect the performance of the pavement [17]. APU exhaust hits the ground at a 45° angle, and the maximum temperature of the surface recorded reached 175 °C after an average exposure of 15 min [17,20]. Figure 1 shows the heat map of an airfield pavement exposed to the exhaust of an F/A-18 Hornet aircraft [17]. The combined effects of the aviation oil spills and elevated temperatures cause surface spalling at airfield pavements. Spalling produces foreign object debris (FOD) in the airfield as small pieces of concrete and aggregates are peeled off from the surface of the pavement. As reported in [17], these FODs pose safety hazards to maintenance crews or severe damage to aircraft engines if sucked in. Depending on the amount of FOD, military airfields may even be declared non-operational and, thus, require quick repair/maintenance. To mitigate damage to the pavement, the concrete needs to exhibit resistance to repeated simultaneous exposure to elevated temperatures and engine/hydraulic oils. Previous studies have indicated that pavements can withstand repeated exposure to temperatures up to 200 degrees Celsius. However, it has been observed that at elevated temperatures, the presence of oil-soaked pore pressure can lead to a reduction in the durability of the concrete, emphasizing the importance of addressing this issue to enhance pavement performance.

This study investigates the influence of LWA on spalling resistance of concrete repeatedly exposed to simulated airbase conditions. Although some studies on the performance of lightweight aggregate concrete exposed to high to very high temperatures are available [15,21], none of the previous studies investigated the combined effects of chemicals and high temperatures on the durability of LWAC. Based on the comprehensive research, residual mechanical and thermal properties and spalling behaviour of LWAC are reported. It can be noted that this study will facilitate selecting the suitable coarse aggregate type for military airfield pavement construction to minimize surface spalling. Furthermore, industries such as food processing and heavy-duty equipment maintenance bays, where floors are often exposed to various oils and heat, can also benefit from the study’s outcomes by minimizing damage and extending the longevity of concrete.

Four different lightweight aggregates—pumice, perlite, lytag, and crushed brick—were chosen to produce concrete specimens for this experimental investigation. Ordinary Portland cement (OPC) was used as a binder for each coarse aggregate type. A set of concrete specimens with basalt aggregate was prepared as the benchmark. The residual mechanical properties of the exposed concrete in terms of indirect tensile strength, stress–strain behaviour, compressive strength, spalling, thermal properties, and microstructural conditions were evaluated and revealed in the present study.

## 2. Materials and Method

### 2.1. Materials

**Figure 2:**
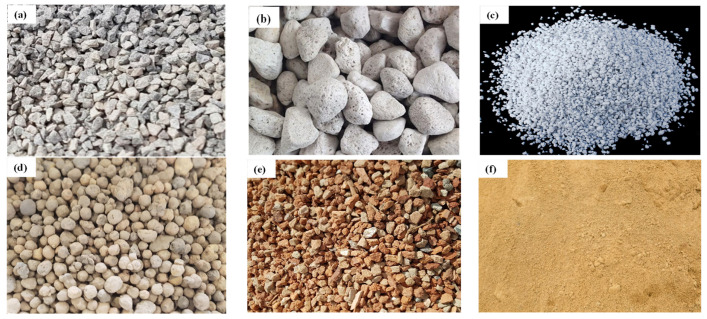
Types of coarse and fine aggregates: (**a**) Basalt. (**b**) Pumice. (**c**) Perlite. (**d**) Lytag. (**e**) Brick chips. (**f**) River sand.

Australian general-purpose cement (AS 3972) was used for the preparation of concrete specimens [22]. Table 1 shows the chemical analysis of LWA obtained by the XRF analysis. River sand (fineness modulus of 2.60) was used as a fine aggregate. The specific gravity and the water absorption capacity of sand were 2.62 and 0.54%, respectively. Local coarse aggregate (basalt) of a maximum size of 10 mm was used for preparing control concrete specimens. The water absorption capacity, specific gravity, and dry-rodded unit weight of basalt were 0.34%, 2.66 and 1500 kg/m^3^, respectively. Pumice, perlite, lytag (PFA based) and crushed brick were used for preparing the LWAC samples, as shown in Figure 2. Ordinary tap water was used to cast concrete samples. ADVA 650 super plasticizer, which is a modified synthetic carboxylated polymer, was used to enhance the workability of concrete.

### 2.2. Concrete Mixing and Specimen Preparation

The mix ratio of the control and LWAC specimens is presented in Table 2. For mixing and compaction, a rotary drum mixer machine and tabletop vibrator were used. LWAs were pre-soaked to maintain the desired level of workability. Samples were cured in a fog room for four weeks at 23 ± 1 °C and relative humidity (RH) > 90%. Cylinder specimens (100 mm × 200 mm) were cast to determine the residual mechanical properties. After 28 days of fog room curing, the mechanical and thermal properties tests were conducted. All other residual tests were conducted after every 20 cycles of exposure to the conditions. In all cases, an average of three samples of data were recorded.

### 2.3. Thermal and Chemical (HC Fluids) Exposures

**Figure 3:**
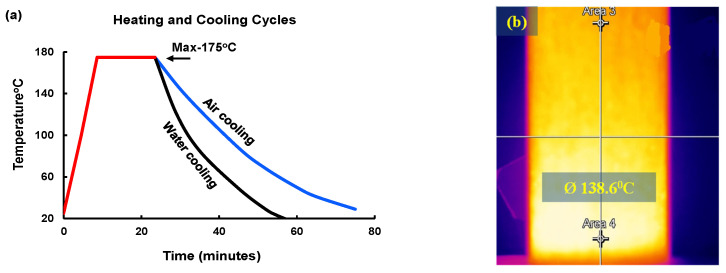
(**a**) Heating and cooling cycle for specimens. (**b**) Heat map of the sample during air cooling (Dark colors on the surface indicate cooler temperatures, while bright colors signify hotter temperatures).

The concrete samples were repeatedly exposed to high temperatures only and were exposed to the coupled effect of HC fluids and high-temperature cycles simultaneously. As reported [17,19,20], military airfield pavements are often soaked with different aviation oils such as aviation fuel, hydraulic oil, and engine oil. Therefore, in this experiment, jet fuel (F-34 kerosene grade), AeroShell Fluid 31 and AeroShell Turbine Oil 500 were procured and mixed in a 1:1:1 ratio and sprayed on the concrete specimens. For thermal exposure, concrete samples were heated in an electric oven at 175 °C for 15 min [17]. Figure 3 shows the duration of a thermal cycle used in the study. After the heat exposures, the concrete samples were kept outdoors to cool down to ambient temperature. Moreover, water was sprayed on the surface of the concrete samples once a week to simulate rainfall effects on the samples. Oil spraying, heating and cooling were cyclically carried out until surface spalling occurred. The concrete samples were exposed to aviation oils and high thermal cycles daily except weekends over the 4–6 months. The notations 0, 20, 40, 60 and 80 denote the number of high-temperature or combined high-temperature and HC fluid exposures, as mentioned in the individual cases.

### 2.4. Tests

#### 2.4.1. Residual Mechanical Properties Test

As per AS 1012.9-2014 [23], LWAC samples were tested for initial and residual compressive strength at intervals of 20 cycles. A universal hydraulic testing machine was used to determine the compressive strength of concrete with a loading rate of 0.3 MPa/s.

#### 2.4.2. Stress–Strain Test

A Technotest (manufacturer, Manila, Philippines) compression testing machine, closed-loop, servo-controlled, with a capacity of 3000 KN, was used for the stress–strain test. A compressor meter with two displacement transducers with a gauge length of 100 mm was directly attached to the test sample to monitor strain. For each specimen, the displacement between the machine plates was also monitored. During the test, a strain rate of 0.07 mm/min was maintained.

#### 2.4.3. Determination of Thermal Properties

The specimens’ specific heat and thermal conductivity were measured after 0, 40, 60 and 80 cycles of exposure. As per ASTM C518 [24], a Netzsch (manufacturer, Selb, Germany) Lambda 446 heat flow machine was used to test 150 mm × 150 mm × 25 mm size samples for specific heat and thermal conductivity. While measuring the specimen’s thermal properties, a 20 °C temperature gradient difference between the two plates was maintained. Two heat flow sensors were attached to the plates to measure the heat flow into the material and out of the material.

#### 2.4.4. Fourier Transform Infrared Spectroscopy (FTIR) Analysis

For FTIR analysis, samples were taken from the top surface up to a depth of 25 mm, which previous researchers reported as the maximum spalling depth [19,25]. A ball mill machine was used to pulverize the collected samples. Then, the FTIR spectrum of each powder sample was obtained. Before each scanning, the background spectrum of the laboratory environment was scanned. The spectra of all samples were obtained by 16 scans using wavelengths between 3800 cm^−1^ and 650 cm^−1^ at the resolution of 4 cm^−1^. Spectra were analysed to understand different covalent bonds, functioning groups, and the decomposition of compounds.

#### 2.4.5. Thermogravimetric (TG) and Differential Scanning Calorimetry (DSC) Analysis

DSC and TG tests were conducted after 0, 40, and 80 cycles of HC fluid and high-temperature exposure. The concrete samples were crushed, milled, and sieved beforehand. NETZSCH STA 449C Jupiter (Gerätebau GmbH, Selb, Germany) was used for both the TG and DSC tests simultaneously. At a constant heating rate of 10 °C/min, the powdered specimens were heated from 20 °C to 800 °C in an inert nitrogen environment to collect the DSC and TG spectra. The derivative thermogravimetry (DTG) technique was used to find the exothermic and endothermic reaction temperature corresponding to the transformation due to heating. Also, the thermogravimetric technique measured the mass loss due to the decomposition of minerals.

#### 2.4.6. Microstructural Investigation

Samples collected from the top surface (25 mm depth) of LWAC were used for microstructural analysis. The LWAC sample microstructures in the original condition and after 80 cycles of HC fluid and high-temperature exposure were analysed by a Zeiss Axio Imager 2 optical microscope. This machine enabled us to study the microcrack and void development in the samples. The LWAC microstructure was analysed using two main techniques: a scanning electron microscope (SEM) and direct observation with an optical microscope. Combining results from both methods allows a better understanding of the morphology of the aggregate, interfacial transition zone (ITZ) cracks, composition, microcrack development and propagation.

## 3. Result and Discussion

### 3.1. Residual Mechanical Properties

#### 3.1.1. Compressive Strength

**Figure 4:**
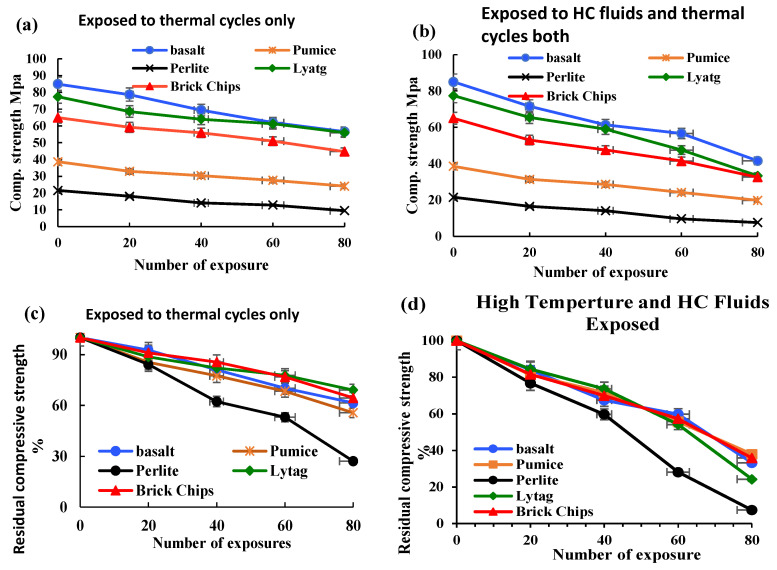
Residual compressive strength. (**a**) High-temperature-exposed LWAC. (**b**) HC fluid- and high-temperature-exposed LWAC; % of residual compressive strength retained. (**c**) High-temperature-exposed samples only. (**d**) HC fluid- and high-temperature-exposed LWAC samples.

The residual compressive strength of LWAC samples was tested at an interval of 20 cycles, as shown in Figure 4. After every 20 cycles of exposure, three concrete cylinders from each group were tested for compressive strength. Due to frequent exposure to HC fluids and high temperatures, all samples lost a significant amount of their initial compressive strength. The initial compressive strength of control, brick and lytag aggregate concrete was in the range of 65–80 MPa. However, the pumice concrete sample and the perlite concrete had compressive strengths of 39 and 22 MPa, respectively. For only high-temperature-exposed samples, the control sample lost 33.40% of the initial compressive strength; similarly, strength losses of 31.20% and 27.6% were recorded for brick and lytag specimens, respectively. Due to lower density, higher porosity and lower stiffness, pumice and perlite LWAC samples suffered significant strength losses of 48.70% and 55.80%, respectively.

The control sample lost 51.10% of the initial compressive strength for combined HC fluid and high-temperature exposures. Under a similar condition, pumice and brick concrete specimen strength losses were 48.70% and 49.80%, respectively. However, lytag and perlite LWAC samples suffered significantly; they lost 57% and 64.4% of initial compressive strength, respectively. The simultaneous effect of HC fluids and thermal cycles is substantially higher than the single effect of high thermal cycles. Also, most of the LWAC showed a lower residual compressive strength than that of the control concrete. Based on the compressive strength test results, lytag and brick LWAC performed much better than pumice and perlite LWAC under the combined action of the repeated high-temperature and HC fluid exposures.

#### 3.1.2. Indirect Tensile Strength

**Figure 5:**
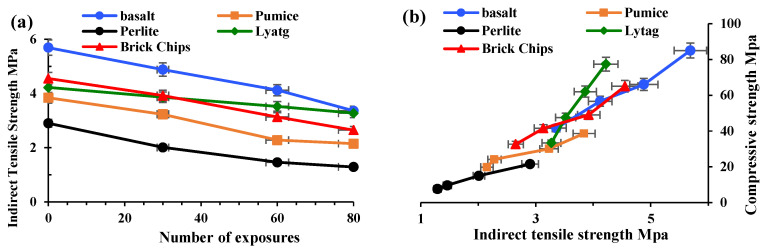
Indirect tensile strength relationship. (**a**) Tensile strength vs. number of exposures to high temperatures and HC fluids. (**b**) Tensile strength vs. compressive strength of concrete after exposure.

Concrete’s tensile strength behaviour indicates its performance under tensile loads and the chances of crack formation in members. Three concrete cylinders from each group were tested for tensile strength. The indirect tensile strength test was carried out at 0, 30, 60 and 80 cycles of exposure, as shown in Figure 5. The indirect tensile strengths of basalt, brick, lytag, pumice and perlite aggregate concrete at 0 exposure conditions were 5.7, 4.55, 4.22, 3.84, 2.9 MPa, respectively. After 80 cycles of high-temperature and HC fluid exposure, the tensile strength losses of basalt, brick, lytag, pumice and perlite concrete were 40.95%, 41.76%, 44%, 55.52%, 22.51%, respectively. The results show that with the increases in the number of heat exposures, the indirect tensile strength is reduced significantly; this agrees with the previous literature [15].

Figure 5b also indicates that both compressive strength and tensile strength decrease with the increase in high-temperature exposures. However, the decrease in tensile strength is more substantial than the compressive strength reduction. These two strengths are closely related, but there is no direct proportionality between them.

#### 3.1.3. Stress–Strain

**Figure 6:**
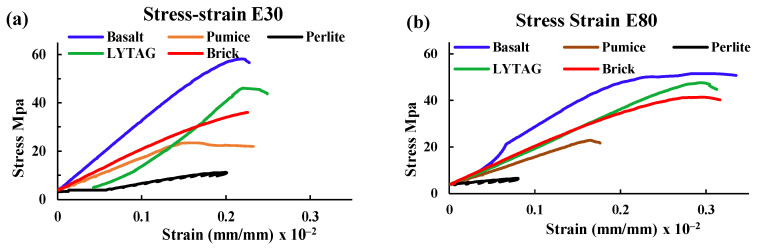
Stress–strain curve of various LWA concrete samples exposed to high temperatures and HC fluids combined. (**a**) After 30 cycles. (**b**) After 80 cycles.

The stress–strain relationship of LWAC after 0, 30 and 80 cycles of combined HC fluid and high-temperature exposures is shown in Figure 6. The stress–strain curve becomes flattened with the increase in the number of exposures. The peak stresses shift downwards and rightwards because of exposures. These phenomena indicate the peak stress decreases and peak strain increases with the increase in exposure number [15]. Therefore, concrete samples reduced stiffness due to the exposure conditions. Concrete with perlite performed the worst compared to other LWA concrete samples. Concrete made with lytag showed better resistance to the simulated conditions compared to concrete samples made with other LWA. Moreover, the concrete with crushed brick aggregates showed considerable resistance to the same conditions even after 80 cycles of exposure. Briefly, regardless of the type of aggregate, exposures of concrete to repeated HC fluids and high temperatures cause a significant loss of compressive strength and an increase in corresponding strain.

Similar to the findings of some other researchers [16,26], this study also believes that the texture, shape and gradation of LWA directly influenced the stress–strain behaviour of concrete. Moreover, the porosity and particle density of aggregates influence the stiffness of concrete; as perlite has higher porosity and lower density, it showed poor performance against the exposure conditions. The reasons for the phenomenon are further investigated by using SEM and optical microscopes and are reported later in the paper.

#### 3.1.4. Concrete Spalling

**Figure 7:**
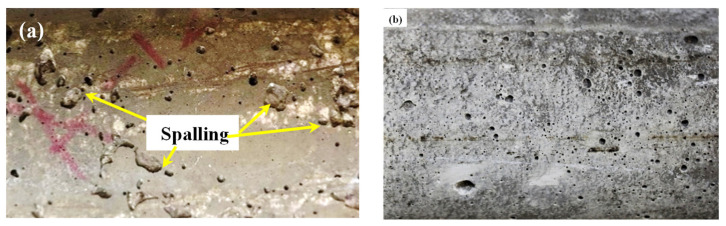

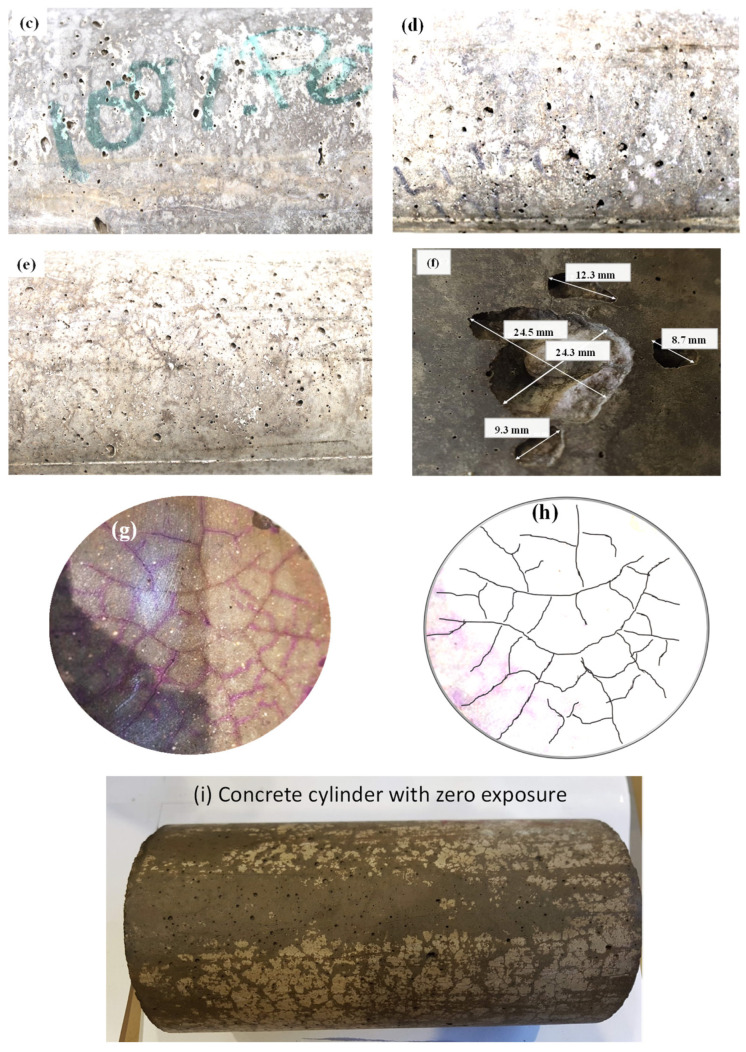
Surface spalling and cracks. (**a**) Control, (**b**) pumice, (**c**) perlite, (**d**) lytag, and (**e**) brick aggregate concrete. (**f**) Details of control spalling. (**g**,**h**) Surface cracks on control samples, and (**i**) concrete cylinder with zero exposure.

Some researchers [27,28,29] reported that spalling in high-strength concrete occurred when subjected to very high temperatures. Generally, up to 200 °C, concrete does not suffer any spalling damage. However, from Figure 7, it is evident that the basalt concrete specimen suffered significant spalling damage due to the recurrent coupled effect of HC fluids and a 175 °C temperature. After 80 cycles of HC fluid and high-temperature exposure, pumice, perlite, and lytag specimens also suffered spalling damage, but the degree of spalling of LWAC was less than that of normal concrete. The maximum size of spalling in the basalt aggregate was 24.33 mm × 24.49 mm × 5.6 mm. Under a similar condition, brick LWAC showed no spalling damage. Similarly, LWAC samples exposed to 80 cycles of only high temperatures exhibited no significant spalling. When exposed to only high temperatures, the dense microstructure of normal-weight concrete (NWC) prevents moisture from escaping, thus causing cracking in concrete. When exposed to the combined effect, HC fluids trigger strength degradation by forming detrimental salts [18,19,30], and heat causes the formation of cracks; thus, the combined effects of chemicals and high temperatures cause the spalling. Overall, LWAC samples did not suffer significant spalling because of their porous microstructures, as vapour pressure was released immediately when heated.

#### 3.1.5. Mass Loss

**Figure 8:**
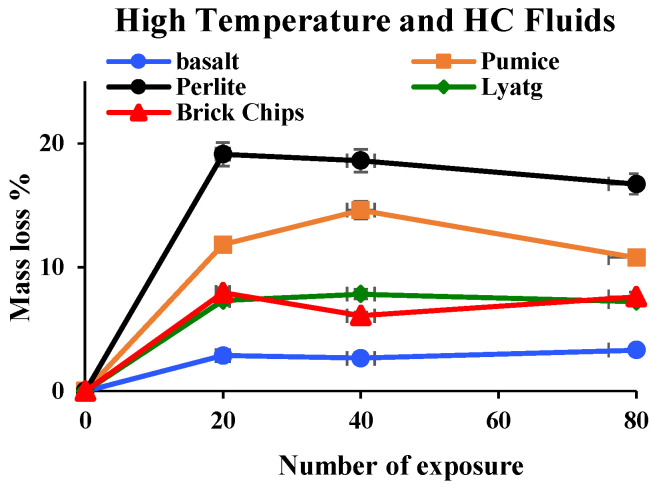
LWAC sample mass losses after exposures to repeated HC fluids and high temperatures.

Figure 8 shows the mass loss percentage of LWAC samples after exposure. Within the first few cycles, samples lost their initial free water entirely due to frequent high heat exposures. The mass loss curves flattened after 20 cycles of exposure to high temperatures and HC fluids, suggesting that the concrete matrix lost its initial free water. Maximum water losses of LWAC samples after the first 20 cycles were 19.13%, 11.83%, 7.96% and 7.31%, respectively, for perlite, pumice, brick and lytag. Pumice and perlite LWACs’ water losses were higher because they held more free water due to higher permeability. Khalifa et al. [31] also reported that the concrete segment’s permeability between the drying dehydrating front and the heated face controls the mass loss kinetics. Normal-weight aggregates (NWA), such as basalt, having higher density and lesser porosity, hold less pore water, thus releasing only 2.87% of water due to heating.

Due to repeated exposure to a temperature of 175 °C, extra water evaporation occurs; when this free water is lost, mass loss continues due to decomposition of cement elements and the release of chemically bound water. After 80 cycles of exposures, the mass losses for basalt, pumice, perlite, lytag and brick were 3.71%, 4.44%, 7.72%, 4.98% and 3.95%, respectively. As the mass loss could have been due to the decomposition of mineral compounds in concrete, the mass loss phenomenon due to cement element decomposition was further investigated by TG analysis later in the paper.

### 3.2. Thermo-Mechanical Properties

#### 3.2.1. Thermal Conductivity

**Figure 9:**
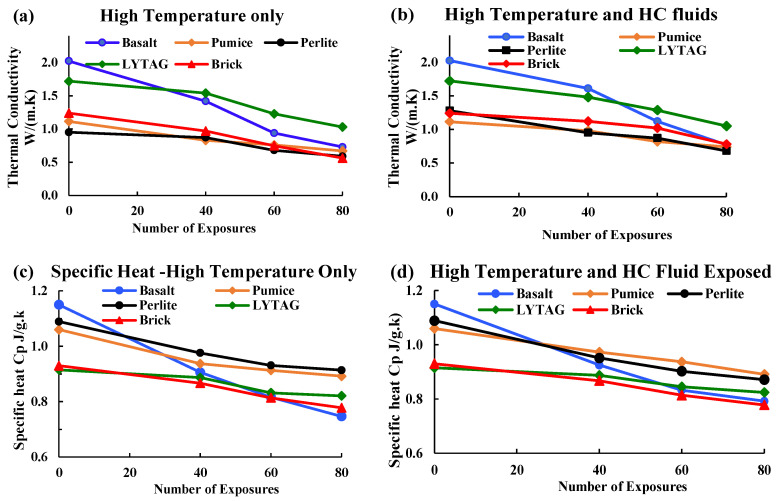
(**a**) Thermal conductivity of samples exposed only to heat. (**b**) Thermal conductivity of samples exposed to HC fluids and heat. (**c**) Specific heat of heat-exposed samples. (**d**) Specific heat of samples exposed to HC fluids and heat.

Thermal properties of concrete depend on many factors, including material mix proportion, types of aggregate, aggregate structure, density, porosity and degree of crystallisation [32,33]. At ambient temperature, the thermal conductivity of NWC is reported to be in the range of 1.4–3.6 W/m·K, and it changes with an increase/decrease in temperature [34]. LWAC shows lower thermal conductivity due to its high porosity and entrapped air’s low thermal conductivity. Thus, replacing NWA with LWA affects concrete porosity and its thermal conductivity [35]. Figure 9 shows the thermal conductivity of LWAC subjected to high temperature only and simultaneously exposed to high temperatures and HC fluids. Samples’ thermal conductivity was measured at 30 °C at different exposure conditions, for example, after 0, 40 and 80 cycles of exposure.

The thermal conductivities of basalt, lytag, brick, pumice and perlite aggregate concrete were 2.02, 1.72, 1.24, 1.11 and 0.95 W/m.k, respectively. From the varying initial thermal conductivity values, it is understood that variation in aggregate structure, porosity and crystallinity affects their thermal conductivity. Thermal conductivities for basalt, lytag, brick, pumice and perlite LWAC samples after 80 cycles of high-temperature exposures were 0.73, 1.03, 0.56, 0.67 and 0.59 W/m.k, respectively. With an increase in high-temperature and HC fluid exposures, the thermal conductivity of concrete reduces gradually. Basalt and brick aggregate concrete suffered 63.91% and 54.68% loss of thermal conductivity after 80 cycles, whereas other LWAC suffered 37–40.12% losses only from original conditions.

In summary, the basalt aggregate’s thermal conductivity value was much higher than that of the LWA. This may be due to the dense microstructure and higher crystallisation of NWA concrete. Due to repeated exposures to the coupled effect of HC fluids and high temperatures, these samples had higher thermal conductivity than other samples, because fluid exposure increases the pore water, which is thermally more conductive than pore air. In all concrete samples, thermal conductivity decreased significantly with increased exposures due to crack growth and propagation.

#### 3.2.2. Specific Heat

The higher specific heat of concrete helps to increase its temperature stability. Usually, the specific heat of different concrete aggregates at ambient temperature varies between 0.84 and 1.8 J/g·K. LWAC samples tested during this test had an initial specific heat from 0.92–1.2 J/g·K. Various physical and chemical transformations at higher temperatures directly influence LWAC samples’ heat properties. TG analysis (Section 3.3.3) shows that at about 150 °C, concrete lost almost all free water and crack development started, influencing heat properties. Figure 9 illustrates the variation of specific heat of basalt and LWAC samples exposed to high temperatures only and combined exposure to the high temperatures and HC fluids.

The graph shows that, initially, basalt aggregate has higher specific heat than other LWAC samples. With an increase in the number of exposures to the combined effects of high temperatures and HC fluids, the specific heat reduces gradually, especially for basalt aggregate. Though basalt, pumice, and perlite aggregate concrete had almost similar specific heats, after 80 cycles of high-temperature exposures, basalt concrete’s specific heat reduced by 35%, whereas other LWA concrete lost around 10–16% only, as shown in Table 3. After 80 cycles of exposure to HC fluids and high temperatures, specific heat reduction in basalt, pumice, perlite, lytag and brick aggregate concrete samples was 31.13%, 15.94%, 20.01%, 9.94% and 10.26%, respectively.

In brief, in the original condition, LWAC has lower thermal conductivity than NWC, but after 80 cycles of high-temperature exposures, LWA’s specific heat was higher than that of basalt aggregate. It implies that LWA show much more stable behaviour under high-temperature exposure than basalt. Nevertheless, considering mechanical strength and thermal properties, brick aggregates will be more suitable for concrete uses that need higher mechanical strength and good thermal properties.

### 3.3. Thermochemical Properties

#### 3.3.1. FTIR Spectrums of HC Fluids

**Figure 10:**
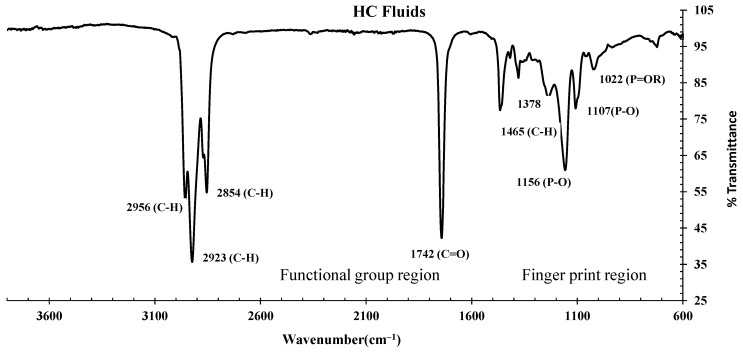
The FTIR spectrum of hydrocarbon fluids used.

Figure 10 represents the FTIR spectrum of the HC fluid used in this experiment, which is primarily made by mixing jet fuel, hydraulic fluids, and engine lubricating oil in equal proportions. The C-H bond’s stretching vibration was represented by three prominent peaks at 2956, 2923 and 2854 cm^−1^, which dictates the presence of alkanes [17,18,19]. The ester of fatty acids represented by the stretching vibration of the C=O bond was detected with a major peak at a wavenumber of 1742 cm^−1^ [19,30]. In the fingerprint region, medium to weak peaks at a wavenumber of 1465, 1378 and 731 cm^−1^ represent the aromatic alkenes, -CH_2_-, -CH_3_-, representing the bending vibration of the C-H bond [19,30]. Peaks at 1156, 1107 and 1022 cm^−1^ dictate the P=O bond of phosphonate, phosphate ester/phosphoric acids [36,37].

Based on the FTIR analysis of HC fluids used in this experiment, the phosphonate, phosphine oxide, phosphate and esters of fatty acids were identified as part of lubricating oils. Similarly, free alcohol/phenol, alkanes, phosphate esters and esters of fatty acids were present in the hydraulic fluid. This oil also contains long-chain hydrocarbons of -CH_3_-, -CH_2_-, alkenes and aromatic compounds.

#### 3.3.2. FTIR Analysis of LWAC Samples

**Figure 11:**
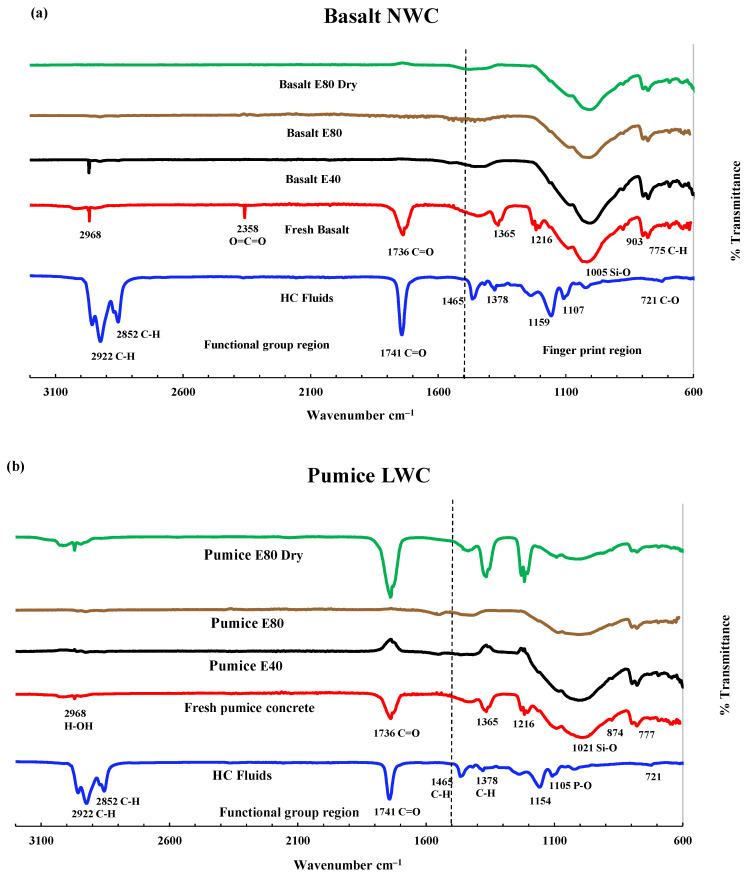
FTIR analysis. (**a**) Basalt NWC. (**b**) Pumice LWAC.

The control concrete specimen FTIR spectrum shows sulphate, silicate, carbonate and hydroxide peaks [38]. Figure 11a shows the FTIR spectrum of the control specimen in the original condition, after 40 and 80 HC fluid and high-temperature exposures and 80 cycles of high-temperature exposures only. A small broad peak denotes chemically bound water for H-OH bonds’ stretching vibration at wavenumber 2968 cm^−1^. The carbon dioxide is represented by the stretching mode of C=O by a small weak peak at a wavenumber of 2358 cm^−1^ [39]. The presence of limestone from Portland cement is symbolised by the stretching mode of the C-O bond, detected by a broad medium peak at wavenumber 1365 cm^−1^ and 780 cm^−1^ [38]. Calcium sulphate is represented by the stretching mode of the S=O bond detected at a wavenumber of 1216 cm^−1^ [38]. C-S-H gel and related silicate paste represented by the Si-O bond are seen at a noticeable broad peak of 1005 cm^−1^ [38,40]. Finally, the quartz components’ crystalline phase was detected at the peaks at 693 and 646 cm^−1^ [20,25,27].

**Figure 12:**
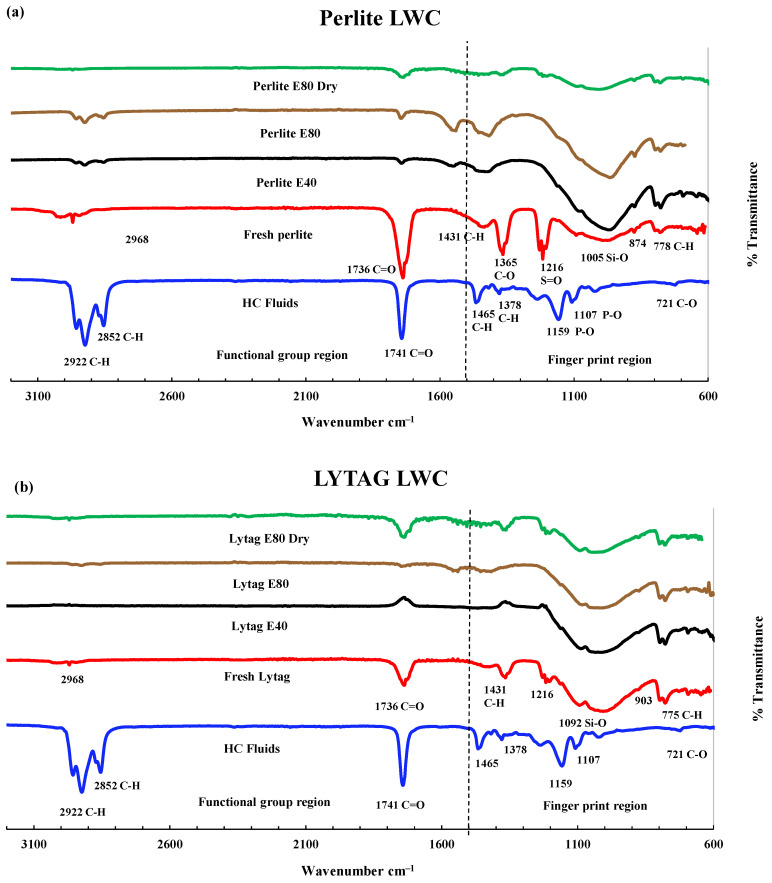

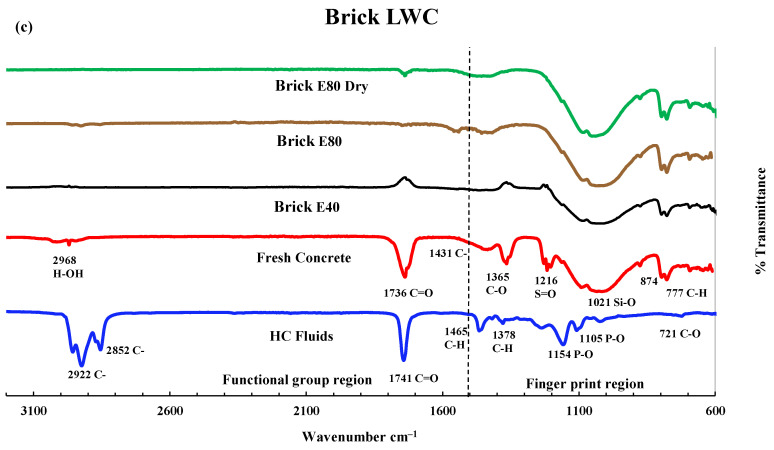
FTIR analysis. (**a**) Perlite LWA concrete. (**b**) Lytag LWA concrete. (**c**) Brick LWAC.

Significant changes were observed in pumice and perlite at 0 and 80 cycles in peak height, shifting of position or total disappearance due to repeated exposure to high temperatures and HC fluids. Figure 12a, in the second and third spectra, shows the FTIR spectrum of basalt concrete exposed to high temperatures and HC fluids after 40 and 80 cycles. It was noticed that, due to repeated high-temperature and HC fluid exposures, the basalt concrete peak for hydroxyl ion (-OH) of Ca(OH)_2_ was destroyed by the chemical compounds of HC fluids. The alkane-related C-H bonds’ stretching vibration was detected at 2981 and 2883 cm^−1^ [19,30]. Usually, ordinary cement does not contain any alkane molecules; HC fluids contributed alkanes. Changes are observed in the silicate absorbance bands in the 1200–800 cm^–1^ wavenumber region [19,30].

No peaks for the esters of fatty acids were detected in the basalt concrete cylinders. Calcium salt was produced due to a chemical reaction between calcium hydroxide and fatty acids [19,30]. Similarly, as seen in Figure 12, after 80 cycles of high-temperature exposures of pumice, perlite and lytag LWAC, peaks at 1216, 1365, and 1735 cm^−1^ are still available; due to non-exposure to HC fluids, they are not consumed by alkanes or esters of HC fluids. Only dehydration of concrete occurs in samples exposed to high temperatures.

#### 3.3.3. Thermogravimetric Analysis

**Figure 13:**
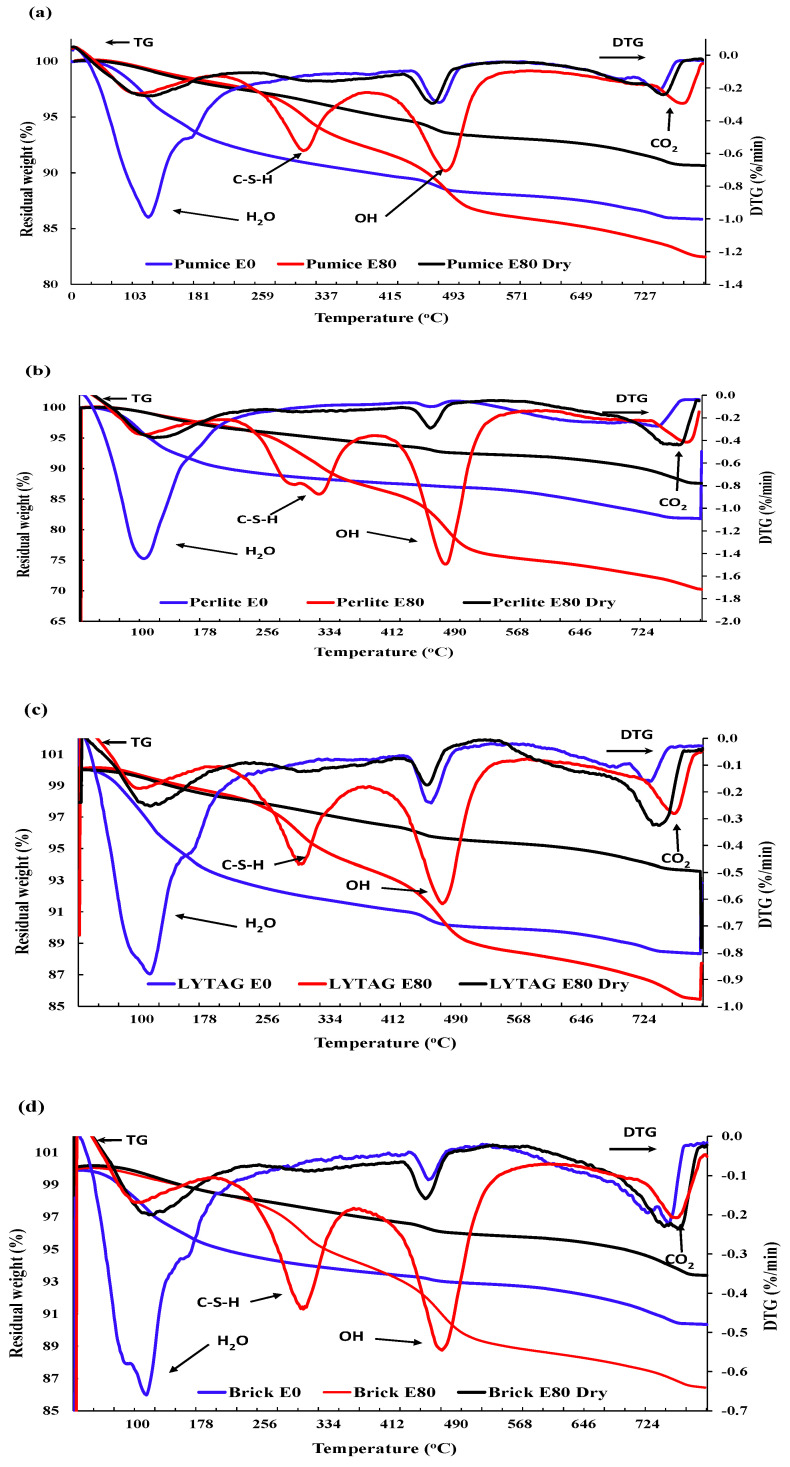
TG and DTG analysis of LWAC exposed to HC fluids and high temperatures. (**a**) Pumice. (**b**) Perlite. (**c**) Lytag. (**d**) Brick.

Figure 13 shows the thermogravimetric (TG) test result for LWAC samples exposed to recurrent HC fluids and high temperatures. The TG curve of the original concrete showed three rapid weight-loss sections. The peaks between 20 and 200 °C represent weight losses due to the evaporation of parts of the bound water and free water; Fares et al. [41] also reported that the free water was eliminated at 120 °C. Gypsum starts decomposing at 182 °C along with ettringite [42] and carboaluminate hydrates and causes mass loss. This sharp mass loss in the 30–200 °C temperature range was evaluated to be about 3.19%, 7.04%, 9.84%, 6.67% and 4.93%, respectively, for basalt 0, pumice 0, perlite 0, lytag 0 and brick 0. Repeated exposure to HC fluids and high-temperature cycles caused a reduction in mass loss. After initial exposure, excess and bound water almost dries up. With the increase in high-temperature exposures, the mass loss above 600 °C increases.

The DTG graph shows that after the initial rapid decline, the second weight loss in the range of 200–350 °C was due to C-S-H gel decomposition and bound water release. The most significant weight loss is observed from 350–600 °C, where portlandite starts disintegrating until completion of the process at around 480 °C [43]. The mass loss after 80 cycles was 10.95%, 5.94%, 5.04% and 4.8% in perlite, pumice, lytag and brick, respectively. This significantly higher value is only observed in samples exposed only to HC fluids, which may be due to cement compounds’ chemical reactions with the element of aviation oils. However, after 400 °C, mass loss was significantly higher for ordinary concrete control specimens than for LWAC samples. In the range of 700–800 °C, the mass loss was due to the decomposition of C-S-H (II) into wollastonite and larnite and the release of carbon dioxide, which accounts for 4.75%, 7.14%, 3.9% and 3.39% of mass loss in pumice, perlite, lytag and brick 80-cycle samples, respectively.

In brief, the porous microstructure of pumice and perlite has allowed more absorption of pore water. Thus, pumice and perlite aggregate exposed repeatedly to high temperatures and HC fluids suffered more mass loss than brick and lytag aggregates.

#### 3.3.4. DSC Analysis

**Figure 14:**
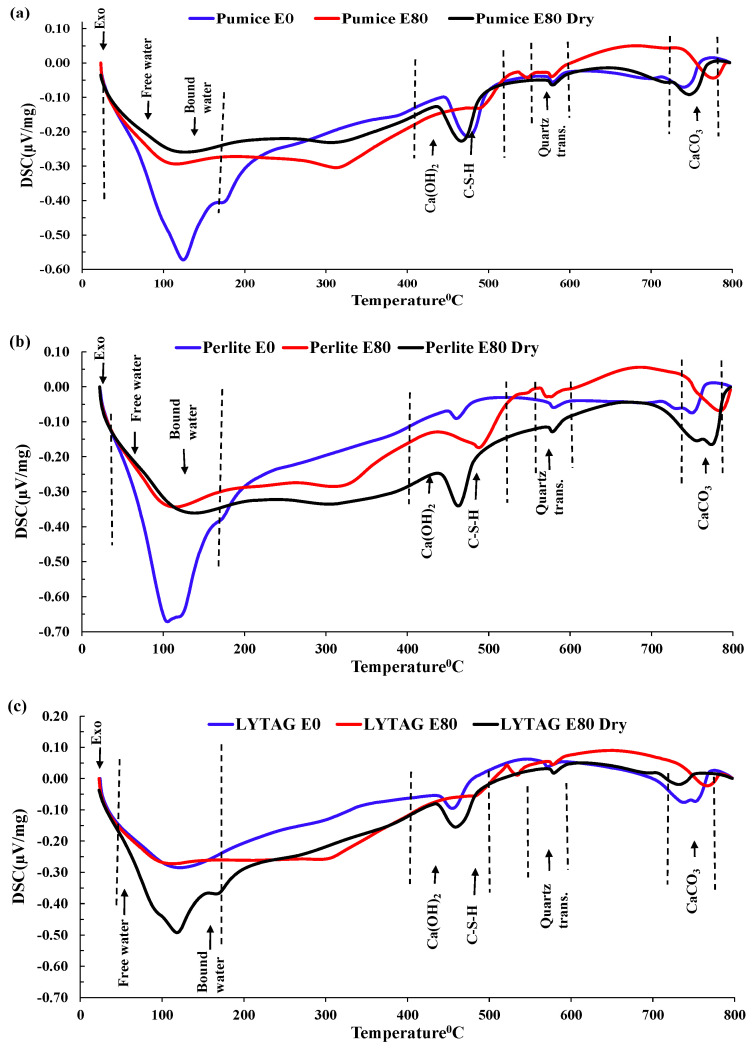

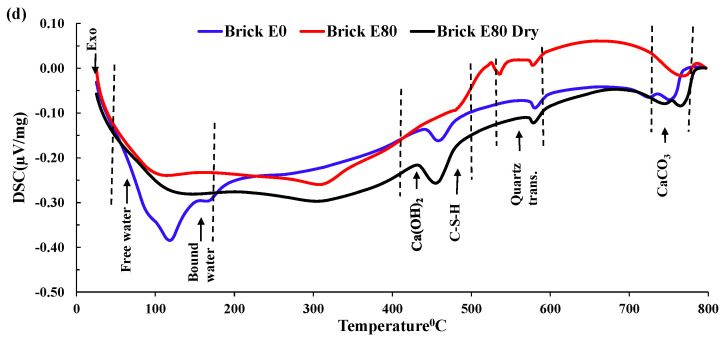
DSC of LWAC exposed to high temperatures and HC fluids (dotted lines indicates the corresponding temperature). (**a**) Pumice. (**b**) Perlite. (**c**) Lytag. (**d**) Brick.

In a DSC analysis of LWAC samples repeatedly exposed to high temperatures and HC fluids, we observed four distinct exothermic peaks at 30–150 °C, 400–500 °C, 550–570 °C and 700–750 °C, as shown in Figure 14. From 50–150 °C, the double peaks indicated significant mass loss due to the partial loss of evaporable water and chemically bonded water from C–S–H gel and ettringite hydrates [18,19,30]. In the 100–300 °C temperature range, rapid water migration caused microcracks in specimens, triggering the rapid degradation of residual compressive strength.

According to Khoury [44], the continuous dehydration of C–S–H between 200 °C and 300 °C caused a slight variation of heat flow in this range. However, several other researchers [45] reported that the breakdown of tobermorite gel and water loss from pores of hydrates caused heat flow variation in the 200 °C to 400 °C temperature range. Around 430–450 °C, portlandite decomposed into free lime (dehydroxylation) with a significant peak. At 573 °C, the solid-state phase transformation of quartz-α into quartz-β occurs with an enlargement/cracking in some coarse aggregates. Similar to previous findings, we also detected the decomposition of C-S-H gel and the formation of β-C_2_S from 600–700 °C. Between 700 °C and 800 °C in the exothermic phase, the decomposition of the CaCO_3_ caused the release of CO_2_ [36,44], although insignificant mass loss occurred. Repeated exposures to HC fluids and higher temperatures caused some phases’ partial or total disintegration, because several peaks disappeared or reduced/shifted to a higher temperature. Samples with higher porosity, like pumice and perlite, have higher peaks and give up more water in the initial temperature range.

In brief, in the 100–300 °C temperature range, rapid water migration develops microcracks in specimens, leading to the rapid degradation in residual compressive strength. DSC confirms that evaporable and chemically bound water was jettisoned gradually according to their binding forces. The evaporation of free and bound water caused an increase in the concrete’s porosity. These chemical and physical changes resulted in changes to the LWAC samples’ residual mechanical properties and thermal properties.

### 3.4. Morphology and Microcracks—Voids

#### 3.4.1. Morphology of Aggregates

**Figure 15:**
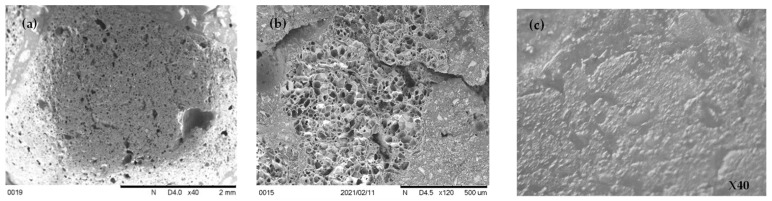
Morphology of different LWA. (**a**) Lytag. (**b**) Perlite. (**c**) Brick.

Figure 15 shows the micrographs of the lytag, perlite and brick LWA concrete obtained using SEM. The microstructures of the various LWA had significant differences among them. The microstructures of basalt, brick, pumice, lytag and perlite aggregates vary from solid to porous and significantly affect thermal and mechanical properties, as seen in previous sections. The diameter of the pores of LWA varied from 10–70 μm. Generally, the pores inside the LWA were independent of each other and separated from the outer surface; however, the perlite aggregate pores were connected to outer surfaces. Additionally, SEM images revealed that LWA surface morphology was uneven, unlike the NWA; thus, better bonding between the aggregate and cement paste occurred and improved the mechanical interlocking action.

#### 3.4.2. ITZ, Microcracks and Voids

**Figure 16:**
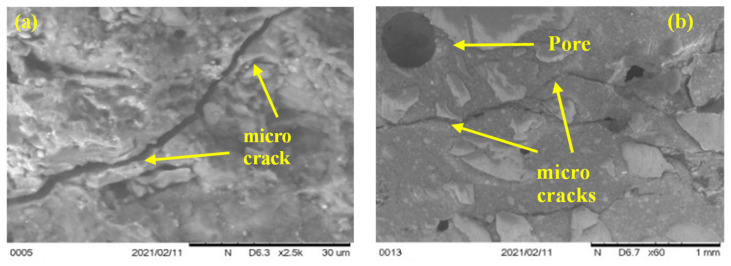

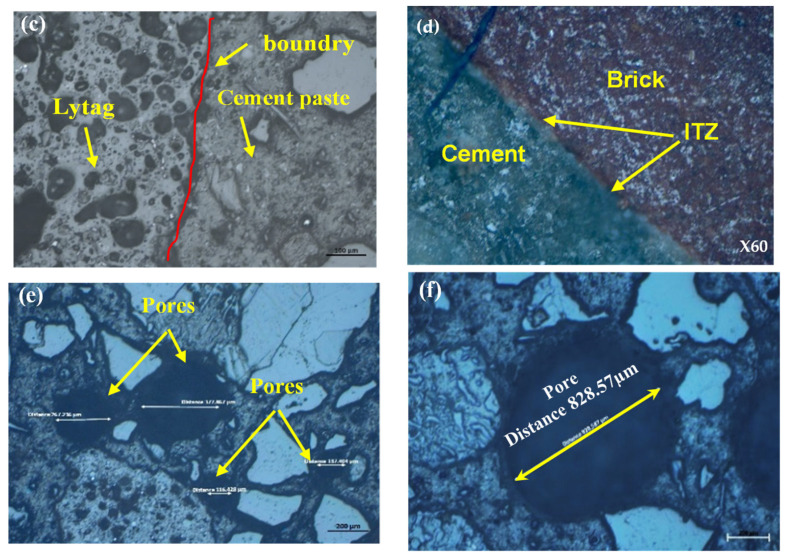
ITZ SEM micrographs for LWAC and NWC: (**a**) Brick sample microcrack (×2.5 k). (**b**) Multiple cracks in pumice aggregate (×60). (**c**) ITZ between LYTAG and cement paste (×20). (**d**) ITZ between brick and cement paste (×20). (**e**) Multiple pores in pumice concrete. (**f**) Large irregular pore in basalt concrete.

SEM and optical microscopic investigation were carried out after 0 and 80 cycles of recurrent HC fluid and high-temperature exposure for effects on the ITZ, types of aggregate, and hydration of cement paste. Due to significant differences in the morphology of LWA, the boundary between LWA and cement paste can be distinguished. LWA shown in the above are characterized as having porous interior structures. Clear-cut thermal cracks between the aggregate and the cement paste were found in basalt, pumice and brick aggregates. By magnifying the ITZ between the aggregate and the cement paste by 2.5 k, as shown in Figure 16a, the thermal crack was measured to be approximately 3 μm. Moreover, by SEM micrograph, we found that LWAC bonding between the cement paste and aggregate is much better than between NWA and the cement paste [46].

Basalt aggregates in NWC act as a barrier to thermal microcrack propagation and allow it to spread along with the cement paste–aggregate interface. Moreover, microcracks in LWA start from cement paste then pass through the ITZ into the weak, porous lightweight aggregate. Thus, microcrack propagation helped us to understand the LWA failure mechanism. Like these, thermal cracks develop due to thermal stress development, and the number of cracks increases with the increase in high-temperature exposures. Thermal cracks in perlite aggregate did not follow the ITZ; instead, they followed a weak and porous zone, Figure 16b.

Furthermore, due to the increase in high-temperature exposure, the quantity and extent of thermal cracks increase. A significant increase in air voids was observed in basalt and brick aggregate concrete samples due to aggregate non-porosity. Due to repeated heating and HC fluid exposure, the ejection of water/HC fluid vapor creates numerous irregular air voids, which allow the penetration of HC fluids. The destructive effect of vapour pressure during high-temperature exposure is evident in the form of cracks within the porous aggregates.

The thermal crack analysis indicated that the crack follows the weakest zone in the concrete matrix. If the concrete is made of a lightweight (porous) aggregate, concrete releases water vapour through the porosity of aggregates and results in a minor crack or no crack. In NWC, due to the aggregate’s dense and sturdy microstructure, the thermal crack follows the ITZ between the aggregate–cement paste.

## 4. Conclusions and Recommendation

This study revealed the resistance of various types of LWAC to simulated airfield conditions. Residual mechanical, thermal, chemical, and microstructural properties of LWAC were carefully investigated after repeated exposures to the simultaneous effects of high temperatures and chemicals. The following key conclusions can be drawn from the experimental study conducted:

- LWAC retained a moderate amount of residual mechanical strength after being exposed to repeated high temperatures and HC fluids. After 80 cycles of exposure to the coupled effects of HC fluids and high temperatures, both control and LWAC suffered a significant strength loss. Lytag and brick LWAC showed higher compressive strengths compared to other LWAC types and retained a residual compressive strength like the control specimen.
- The thermophysical properties of concrete are directly linked with the type and strength of the aggregate in concrete. LWAC showed much better thermal performances than NWA concrete under the repeated actions of HC fluids and high temperatures. Among the LWAC specimens used, perlite and pumice concrete specimens showed better thermal performances than lytag and brick aggregate concrete samples due to their porous microstructures.
- Mass loss was prominent for pumice and perlite aggregate concrete due to their higher percentages of porosity. TG and DSC tests showed that the decomposition of cement causes the evaporation of bound water, increasing the mass loss substantially.
- Concrete samples exposed to the coupled effects of HC fluids and high temperatures suffered from spalling damage. Basalt concrete (control) was detected to develop significant spalling. However, pumice, perlite and lytag LWAC experienced relatively lower spalling than did the control specimens. However, crushed brick concrete showed no spalling damage under the same exposure conditions.
- SEM scans revealed that the low porosity in basalt aggregate caused cracking in the cement paste and aggregates at elevated temperatures as the vapour pressure was not able to be released immediately. Therefore, basalt concrete experienced higher heat-induced microcracks compared to other LWAC tested.

In brief, LWA types impact the residual thermo-mechanical properties of concrete. Based on the residual mechanical strength and thermal properties, brick LWAC seems to be a promising material for reducing surface spalling of airfields’ rigid pavements. For further study, concrete samples can be exposed to one-direction (1D) heating, as happens under APU exhaust.

## Figures and Tables

**Table 1:** Qualitative XRF chemical analysis of lightweight aggregate and cement.

Chemical Analysis (% by Mass)	Pumice	Perlite	Lytag	Brick	Basalt	Portland Cement	GPC *
Calcium oxide (CaO)	3.37	2.14	3.44	8.095	4.64	64.5	4.29
Silica (SiO_2_)	71.41	75.64	41.35	56.67	58.24	20.20	60.06
Alumina (Al_2_O_3_)	10.46	10.91	20.89	11.76	15.72	4.8	17.52
Iron oxide (Fe_2_O_3_)	6.70	2.81	23.54	15.03	9.79	3.1	10.68
Sulphur trioxide (SO_3_)	0.86	0.81	1.09	0.44	0.668	2.70	0.901
Magnesia (MnO)	0.21	0.22	0.152	0.30	0.137	1.20	0.228
Titanium oxide (TiO_2_)	0.65	-	1.17	1.07	1.09	0.52	2.15
Potassium Oxide (K_2_O)	6.00	7.40	5.59	5.68	9.13	0.67	3.56
Specific Gravity	1.5	0.30	2.10		2.66	-	-
Moisture %	45	35	15	12.5	4.3	-	-

* GPC—Geopolymer cement.

**Table 2:** Mix design of concrete and dry unit weight of the samples.

Designation of Mixture	Cement (kg/m^3^)	Sand (kg/m^3^)	Coarse Aggregate (kg/m^3^)	W/C Ratio	Superplasticizer (kg/m^3^)	Dry Unit Weight of Concrete (kg/m^3^)
Control	462	792	1012	0.45	0	2323
Pumice	462	714	368	0.45	1.0	1897
Perlite	320	714	407	0.37	5.0	1578
Lytag	280	800	835	0.45	4.8	1909
Crushed Brick	462	750	1035	0.45	3.0	2094

**Table 3:** Loss of % of thermal conductivity and specific heat after 80 cycles of exposures to HC fluids and high temperatures.

Types/Loss	Thermal Conductivity %		Specific Heat %	
	Heat Exposed	HC and Heat Exposed	Heat Exposed	HC and Heat Exposed
Basalt	63.91	61.94	35.04	31.13
Brick	54.68	37.09	16.34	10.26
Lytag	40.12	38.97	7.44	9.94
Pumice	39.86	33.57	15.85	15.94
Perlite	37.96	46.87	16.07	20.01

## Data Availability

Data are contained within the article.
